# Gallbladder neuroendocrine carcinoma: An important differential diagnosis of gallbladder adenocarcinoma

**DOI:** 10.1002/jgh3.12556

**Published:** 2021-05-10

**Authors:** Kenji Ikezawa, Ryoji Takada, Nobuyasu Fukutake, Tomoyuki Otsuka, Shigenori Nagata, Kazuyoshi Ohkawa

**Affiliations:** ^1^ Department of Hepatobiliary and Pancreatic Oncology Osaka International Cancer Institute Osaka Japan; ^2^ Department of Medical Oncology Osaka International Cancer Institute Osaka Japan; ^3^ Department of Diagnostic Pathology and Cytology Osaka International Cancer Institute Osaka Japan

**Keywords:** carboplatin, endoscopic ultrasound‐guided fine‐needle aspiration, etoposide, neuroendocrine carcinoma

## Abstract

Gallbladder neuroendocrine carcinomas (NECs) are a rare but important differential diagnosis of gallbladder cancer. This case report highlights the importance of pathological diagnosis and the efficiency of endoscopic ultrasound‐guided fine‐needle aspiration. Pathological diagnosis should be attempted because the treatment of gallbladder NEC differs from that of gallbladder adenocarcinoma, especially in unresectable cases.

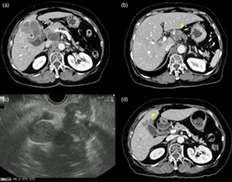

A woman in her 70s, with obstructive jaundice and left periclavicular lymph node (LN) enlargement, was referred to our hospital for further examination. Duke Pancreatic Monoclonal Antigen Type 2 (DUPAN‐2) levels were elevated to 434 U/mL, while carcinoembryonic antigen and carbohydrate antigen 19–9 levels were within the normal range. Contrast‐enhanced computed tomography revealed a hypovascular tumor of the gallbladder fundus with a well‐defined margin accompanied by an intact overlying mucosa, liver invasion (67 mm, Fig. 1a), multiple liver metastases, and multiple paraaortic and hilar LN enlargement (Fig. 1b); these findings suggested a diagnosis of gallbladder adenocarcinoma. However, endoscopic ultrasound‐guided fine‐needle aspiration (EUS‐FNA) with a 22‐gauge needle for a hilar‐enlarged LN revealed a proliferation of small round cells with a high nuclear‐to‐cytoplasmic ratio (Fig. 1c, 2a,b). Immunohistological examinations showed synaptophysin and chromogranin immunopositivities with a high MIB‐1 labeling index (>50%) (Fig. 2c,d), which led to a definite diagnosis of small‐cell neuroendocrine carcinoma (NEC). After a transpapillary placement of a fully‐covered self‐expandable metallic stent (SEMS) for biliary drainage, she underwent six cycles of combination therapy with carboplatin and etoposide, resulting in a partial response with remarkable tumor shrinkage (the maximal diameter of liver invasion reduced from 67 to 29 mm) (Fig. 1d) and spontaneous distal migration of the SEMS. Neuron‐specific enolase also dramatically decreased from 94.9 U/mL before chemotherapy initiation to a normal value. One month after completion of the six cycles of chemotherapy, the tumor relapsed with the emergence of obstructive jaundice. Amrubicin, a second‐line chemotherapeutic agent unfortunately resulted in progressive disease after three cycles.

**Figure 1:**
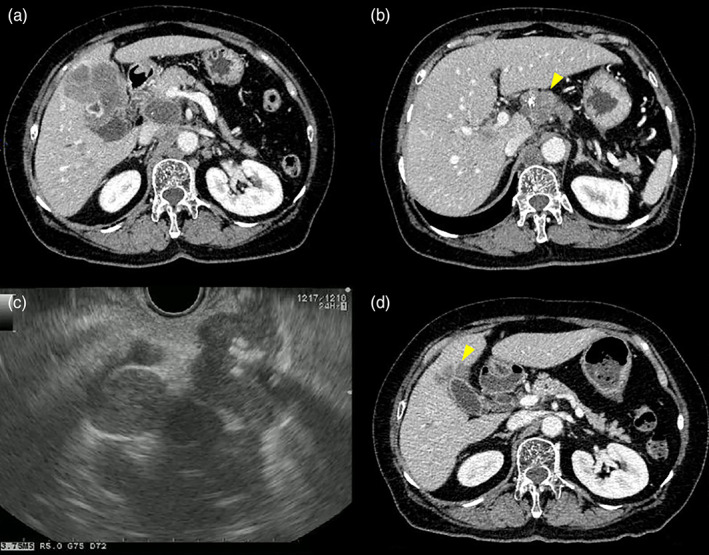
Contrast‐enhanced computed tomography (CECT) images showing (a) hypovascular gallbladder fundus tumor with a well‐defined margin accompanied by an intact overlying mucosa and liver invasion (67 mm), and (b) hilar lymph node (LN) enlargement with calcification (arrowhead). (c) Endoscopic ultrasound image from the stomach showing a hilar‐enlarged LN as a hypoechoic mass with calcification. (d) CECT images showing remarkable tumor shrinkage of liver invasion after six cycles of combination therapy with carboplatin and etoposide (arrowhead).

**Figure 2:**
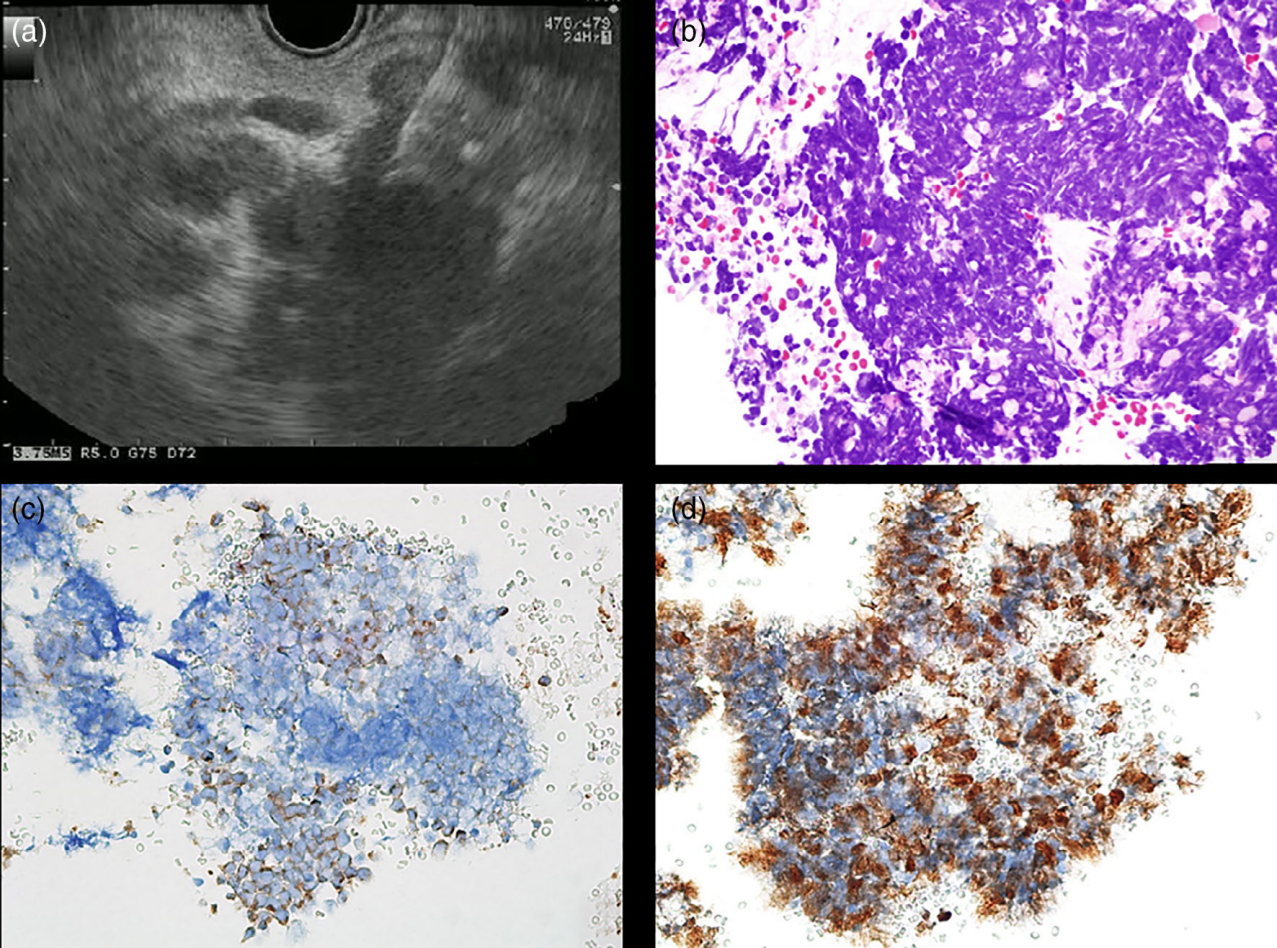
(a) Endoscopic ultrasound‐guided fine‐needle aspiration was performed for a hilar enlarged lymph node. (b) Histological examination with HE staining revealed a proliferation of small round cells with high nuclear‐to‐cytoplasmic ratios (original magnification ×400). Immunohistological examinations showed synaptophysin immunopositivity (c) with high MIB‐1 labeling index (>50%) (d) (original magnification ×400).

Gallbladder NECs are rare gallbladder tumors (0.3–3%) with a poorer prognosis than that of gallbladder adenocarcinoma.^1^, ^2^ Gallbladder NECs account for only 0.2% of all gastrointestinal NECs.^3^ It is generally considered difficult to differentiate gallbladder NECs from gallbladder adenocarcinomas using imaging modalities because gallbladder NECs frequently exhibit hypovascular tumors with LN involvement and liver metastases.^2^, ^4^ However, Kim et al. recently reported that compared with gallbladder adenocarcinomas, gallbladder poorly‐differentiated neuroendocrine tumors frequently exhibited well‐defined margins accompanied by an intact overlying mucosa.^5^ These findings were observed in the present case. It is important to pay more attention to these radiological findings because these findings implicate the possibility of gallbladder NECs. Although elevation of tumor markers raises the suspicion of malignancy, the increase in DUPAN‐2 does not always indicate the existence of adenocarcinoma because it was frequently observed in nonmalignant hepatobiliary diseases.^6^

EUS‐FNA is considered an efficient and safe diagnostic modality for the evaluation of pancreaticobiliary diseases.^7^, ^8^ However, there are very few reports about EUS‐FNA in patients with gallbladder NECs.^9^ In the present case, EUS‐FNA led to a definite diagnosis, which significantly contributed to the selection of appropriate chemotherapy regimens based on the chemotherapeutic treatment of small‐cell lung cancer. Although the proportion of gallbladder NECs is small, this differential diagnosis should be considered because chemotherapeutic regimens are quite different between gallbladder adenocarcinomas and gallbladder NECs.^1^ Additionally, histological samples obtained by EUS‐FNA can be used for cancer multi‐gene panel testing. The importance of pathological examination, including EUS‐FNA, for suspected gallbladder cancer is increasing. EUS‐FNA of enlarged LNs in patients with gallbladder NECs is a promising method to establish a pathological diagnosis because metastatic LNs are larger in patients with gallbladder NECs than in those with gallbladder adenocarcinoma.^5^

In conclusion, gallbladder NECs are a rare but important differential diagnosis of gallbladder cancer. Pathological diagnosis should be attempted because the treatment of gallbladder NEC differs from that of gallbladder adenocarcinoma, especially in unresectable cases.

## Patient consent statement

Written informed consent was obtained from the patient for the publication of this case report and accompanying images.
